# Estimating the Number of Polygenic Diseases Among Six Mutually Exclusive Entities of Non-Tumors and Cancer

**DOI:** 10.3390/ijms252211968

**Published:** 2024-11-07

**Authors:** C. I. Edvard Smith, Jan A. Burger, Rula Zain

**Affiliations:** 1 Department of Laboratory Medicine, Karolinska Institutet, ANA Futura, Alfred Nobels Allé 8 Floor 8, SE-141 52 Huddinge, Sweden; rula.zain@ki.se; 2 Karolinska ATMP Center, Karolinska Institutet, Karolinska University Hospital, SE-171 76 Stockholm, Sweden; 3 Department of Infectious Diseases, Karolinska University Hospital, SE-141 86 Huddinge, Sweden; 4 Department of Leukemia, University of Texas MD Anderson Cancer Center, Houston, TX 77030, USA; jaburger@mdanderson.org; 5 Centre for Rare Diseases, Department of Clinical Genetics, Karolinska University Hospital, SE-171 76 Stockholm, Sweden

**Keywords:** autoimmunity, leukemia, GWAS, SNP, BTK, COVID-19

## Abstract

In the era of precision medicine with increasing amounts of sequenced cancer and non-cancer genomes of different ancestries, we here enumerate the resulting polygenic disease entities. Based on the cell number status, we first identified six fundamental types of polygenic illnesses, five of which are non-cancerous. Like complex, non-tumor disorders, neoplasms normally carry alterations in multiple genes, including in ‘Drivers’ and ‘Passengers’. However, tumors also lack certain genetic alterations/epigenetic changes, recently named ‘Goners’, which are toxic for the neoplasm and potentially constitute therapeutic targets. Drivers are considered essential for malignant transformation, whereas environmental influences vary considerably among both types of polygenic diseases. For each form, hyper-rare disorders, defined as affecting <1/10^8^ individuals, likely represent the largest number of disease entities. Loss of *redundant* tumor-suppressor genes exemplifies such a profoundly rare mutational event. For non-tumor, polygenic diseases, pathway-centered taxonomies seem preferable. This classification is not readily feasible in cancer, but the inclusion of Drivers and possibly also of epigenetic changes to the existing nomenclature might serve as initial steps in this direction. Based on the detailed genetic alterations, the number of polygenic diseases is essentially countless, but different forms of nosologies may be used to restrict the number.

## 1. Introduction

Complex traits were first mathematically interpreted by Ronald Aylmer Fisher in a landmark paper in which the effects of dominance and assortative mating on correlation values were clarified [1], and during the same period, Theodor Heinrich Boveri used groundbreaking experimental approaches to search for the origin of tumors [2]. A hundred years later, next-generation sequencing has enabled a deeper understanding of complex traits from the constantly increasing number of genome-wide association studies (GWASs). This has important implications for polygenic inheritance and risk calculations [3]. GWAS investigations have not only increased the number of recognized polymorphisms in non-cancerous disease but have also resulted in the identification of many novel onco- and tumor-suppressor genes [4,5]. A general challenge for the field of complex diseases is that the rapid augmentation of genetic risk variants by GWASs has outpaced their functional characterization [6]. However, even if this is the case, in Iceland, 4% of the sequenced population were found to have an actionable genotype with implications for their life span [7].

The enormous difficulty in defining diseases is apparent from the description of nosology by Victor A. McKusick published more than 50 years ago, prior to the revolution in molecular diagnostics [8]. In a recent *perspective*, we estimated that there is an almost infinite number of possible diseases considering the influence of genetics, physical insults, and environmental factors, including microorganisms, toxins, temperature, and altitude [9]. In this paper, we detailed the several international efforts which have been instrumental for developing the field. We also introduced the term ‘hyper-rare’ for illnesses affecting fewer than 1 per 10^8^ individuals, because our calculations suggest that this category would outnumber common, rare (fewer than 1 per 2 × 10^3^), and ultra-rare diseases (fewer than 1 per 5 × 10^4^) by orders of magnitude. Thus, the largest number of disease entities, but not the number of affected individuals, is to be found within the hyper-rare group. Owing to the fact that polygenic traits were only briefly outlined in this publication, we here decipher both inherited and acquired polygenic-disease-causing variants in greater depth and discuss how this knowledge is reshaping our definitions of previously established disease entities.

Of note, for such disorders, including cancer, there is not yet the practice to subdivide disease entities according to their genetic variant spectrum. Polymorphisms in neoplasms affect many genes, with the majority being classified as ‘Passengers’, which are neutral, random alterations not contributing to cancer development [10]. Only a unique group of mutated genes acts as ‘Drivers’ that are selected for and which induce tumorigenesis [10]. A third entity is the recently defined ‘Goners’, which are variations selected *against* because they are toxic to the tumor [11,12].

It should be noted that while the concept of Drivers, as genetic alterations causing cancer, is generally accepted, there are examples where drivers are not found among all the tumor samples analyzed [13,14]. However, the absence of identified Drivers does not necessarily mean that they are absent, since not all mutations are readily identifiable.

Hanahan and Weinberg [15] identified eight hallmarks that contribute to cancer and hence are affected by Drivers. These hallmarks include *sustained proliferation signaling*, *evasion of growth suppressors and immune recognition*, *cell death resistance*, *replicative immortality*, *altered metabolism*, and *invasion/metastasis*. For solid tumors, *induction of angiogenesis* is also needed. More recently, *unlocking phenotypic plasticity* was included as an emerging hallmark due to accumulating data showing that the capacity of cancer cells to change their phenotype and state of differentiation is a critical component of cancer progression [16].

What is perhaps the best example of cell plasticity in cancer is epithelial–mesenchymal transition (EMT) engaging transcriptional Drivers belonging to the SNAIL, ZEB, and TWIST families, while requiring an EMT-permissive microenvironment [17,18,19]. Environmental cues, including the tumor microenvironment, could promote neoplastic transformation in the absence of any new Driver mutations, being part of a less well understood developmental process [20]. This relates to the ideas of Conrad Hal Waddington more than 80 years ago, well before the nature of DNA was delineated: “*By such a series of steps, then, it is possible that an adaptive response can be fixed without waiting for the occurrence of a mutation which, in the original genetic background, mimics the response well enough to enjoy a selective advantage*” [21]. To this end, epigenetic mechanisms sustain gene expression and cellular states, while also enabling appropriate responses to developmental or environmental cues. Genetic, environmental, or metabolic insults can induce both restrictive and permissive chromatin landscapes that favor the pathogenesis of cancer and other diseases [22].

To this end, the crucial difference between cancer and non-cancerous disease is that in tumors, and in pre-cancerous conditions, there is hyperplasia in the form of a pauciclonal increase in the number of cells. Thus, during the selection of the fittest neoplastic cells, the balance between growth and cell death is essential for the outcome. Furthermore, although oligoclonal growth normally predisposes an individual to cancer, only some of the affected individuals will develop malignancies. This is likely because the required mutations, epigenetic alterations, or changes in the tumor microenvironment, take a long time to acquire. Examples of such predispositions include monoclonal gammopathy of undetermined significance (MGUS) [23], human-papillomavirus-infected tissues [24], and neurofibromatosis [25]. With regard to acquired genetic alterations, neoplastic transformation is impacted by both inherited and acquired mutations over time. In contrast, most polygenic, non-cancerous disorders are primarily affected by genetic alterations, which are inherited or occur early in life.

As mentioned, apart from genetic variations, acquired epigenetic changes are also of importance for disease [16,21]. Integrating genetics, epigenetics, and gene expression data provides a richer understanding of illness. Thus, the analysis of expression quantitative trait loci (eQTLs) is crucial in the study of polygenic disorders [26]. Until recently, of the more than 200,000 polymorphisms conclusively linked to human complex disease traits through GWASs, the underlying mechanism was mainly unknown [27]. Not long ago, it was reported that the aggregated contribution of *methylated* QTLs (mQTLs), measured as the number of identifiable associations, is larger than that of eQTLs and therefore crucial for the understanding of complex traits [28]. Even more recently, it was estimated that almost 30% of GWAS variants have no detectable relationship to gene expression changes and that eQTLs cluster strongly near transcription start sites, whereas GWAS hits do not [29,30]. In further support of the idea that examination of epigenetics is essential, it was recently also reported that for meningioma, DNA methylation profiling was needed to stratify patients for molecular therapies [31]. Global hypomethylation altering chromatin topology has been suggested to represent a ‘mitotic clock’ corresponding to the number of cell divisions in somatic cells [32]. Hypomethylation also restrains aging cells and limits neoplastic progression [33].

## 2. Subdividing Diseases According to the Maintenance of Cell Numbers

**Figure 1:**
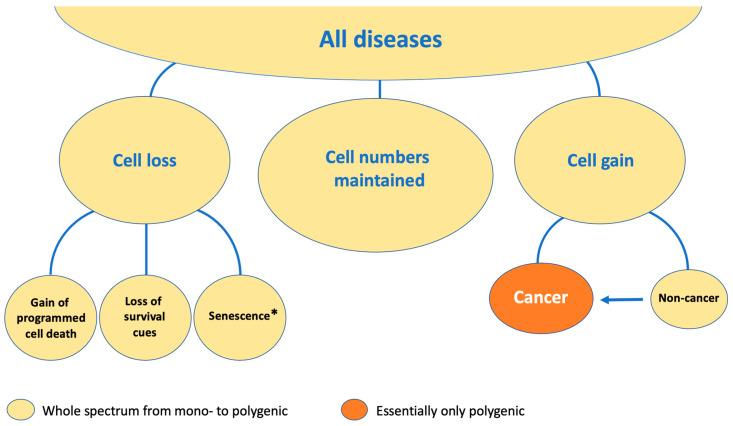
Proposed disease entities as categorized according to whether *cell numbers* are maintained, reduced, or increased. The arrow denotes that oligoclonal expansion of cells in non-cancerous diseases predisposes the individual to neoplastic development owing to the naturally occurring errors taking place during DNA replication. * Senescent cells have essentially lost their ability to divide, causing reduced numbers over time, but the total amount may still be maintained from tissue-resident, replicating, non-senescent cells, albeit not in all disorders [36].

A parameter distinguishing different disease entities is whether cell numbers are maintained, lost, or increased. In contrast to most non-cancerous, polygenic disorders, in tumors, the neoplastic cells continuously increase in number (Figure 1). However, as discussed, non-cancerous diseases may also show increased cell numbers, demonstrating that this variable is not unique to neoplasms (Figure 1).

Nevertheless, even though this parameter does not distinguish neoplasms from non-neoplasms, it is a key feature of diseases, and several disorders with cell gain may eventually develop into tumors. To the best of our knowledge, the subdivision made in Figure 1 has not been made previously, and it shows that only two out of six disease categories demonstrate cell gain. To this end, while there is a net increase, many pre-neoplastic and tumor cells may be lost due to newly acquired deleterious mutations [11,12] because there is a delicate balance between survival and programmed cell death signaling and the influence of environmental cues. Owing to the fact that many mutations occur during replication, disease categorization based on the number of cell divisions might also be informative. Conversely, there are disorders in which cells instead are preferentially lost. Many of the known examples of such non-cancerous diseases in which cell numbers are constantly reduced are monogenic in character. However, it is likely that there exist a plethora of conditions in which polygenic traits underlie the observed loss of cells. Among the known diseases, some lack components necessary for maturation and differentiation (Figure 1). Classical examples are combined immunodeficiencies affecting more than a single lymphocyte lineage [34]. Moreover, in many defects restricted to B lymphocytes [35], there is also a lack of cells because progenitors do not go through the normal steps of development and instead die from programmed cell death.

## 3. Variations Affecting Programmed Cell Death

One of the paths for losing cells is programmed cell death (Figure 1), which is manifested in various forms such as apoptosis, ferroptosis—an iron-dependent pathway causing cell death by a lipid modification called peroxidation—programmed necrosis, and necroptosis—a non-apoptotic mode of cell death that is elicited by ligation of TNF receptor 1 [37]. The prime organism for such studies is the nematode *C. elegans*, for which the fate of all its 1090 cells has been identified, including the 31 that undergo apoptosis [38]. There are several human traits affecting programmed cell death. In the main, most of the known ones are caused by monogenic loss of function, resulting in increased cell numbers [37]. An example is loss of the FAS ligand, which either leads to increased lymphocyte numbers [39] or to systemic lupus erythematosus (SLE) [40]. Loss of apoptosis may also be acquired upon infection with viruses such as human papillomavirus (HPV). This microorganism prevents programmed cell death and profoundly increases the risk for the development of cervical cancer [24].

Various disorders are manifested upon environmental influence, such as drug treatment, an example being the cardiomyopathy caused by doxorubicin. In affected individuals, this therapy induces the degradation of the E3 ligase TRAF2 with subsequent altered NF-κB signaling [41]. Because lack of programmed cell death results in increased cell numbers, some of these conditions are in many respects similar to the situation in cancer and frequently predispose the individual to tumor development.

There are also rare genetic gain-of-function variations affecting programmed cell death (Figure 1), many of which likely result in embryonic lethality. Moreover, in the polygenic form of Alzheimer’s disease, there is evidence of enhanced necroptosis [42]. Selected heterozygous missense mutations in the human *RIPK1* gene instead cause a gain-of-function variation, which prevents RIPK1 enzymatic cleavage upon activation. This leads to autoinflammation, since cleavage inhibits the activation of RIPK3-induced necroptosis [43]. The receptor-interacting protein RIPK1 (also called RIP1) mediates programmed necroptosis and apoptosis, and homozygous loss of function of the corresponding gene results in perinatal lethality and immune system disease [44]. Recently, a connection has been made to prolyl hydroxylation of the hypoxia-inducible factor 1α mediated by the *EGL*-*Nine* homologs–Von Hippel–Lindau tumor-suppressor pathway [45]. It was here reported that inhibiting proline hydroxylation of RIPK1 promotes RIPK1 activation to trigger cell death and inflammation and that this process also involves RIPK3 for the induction of necroptosis. Loss of cells can also be caused by viral infections, such as human immunodeficiency virus (HIV), which induces programmed cell death in CD4^+^ T lymphocytes [46]. Viral infections depend on many factors, and it is therefore considered that a polygenic influence always exists [47].

## 4. Senescence

Senescence (Figure 1) is a cellular stress response triggered by molecular damage. It may be caused by replicative exhaustion due to DNA damage, inhibition of DNA methylases, deranged oncogene activation (also known as oncogene-induced senescence), mitochondrial dysfunction, or treatment with chemotherapeutics [38,48,49]. Senescence can lead to cell death, but senescent cells may also survive long-term, which may negatively influence tissues [36]. A species of particular interest for senescence is the naked mole-rat, which has exceptional longevity and is resistant to age-related physiological decline and diseases. A recent publication suggests that INK4a-retinoblastoma protein-induced cell death likely functions as a natural senolytic mechanism in this species, providing an evolutionary rationale for senescent cell removal as a means to resist aging [50].

## 5. Variations Affecting Atrophy and Hypertrophy

Cells exploit two ways of growth: in size and in number, with the latter preferentially seen during embryogenesis and in cancer (Figure 1). Hypertrophy means increased cell size, whereas atrophy corresponds to either reduced cell size or loss of cells. Myostatin is a secreted ligand belonging to the TGFβ superfamily, whose loss results in increased muscle mass caused both by hypertrophy and hyperplasia [51]. Thus, while myostatin inactivation can induce skeletal muscle hypertrophy, its overexpression, conversely, seems to induce muscle atrophy [52,53]. Cardiac hypertrophy is a leading cause of sudden death and shows polygenic inheritance [54]. Atrophy can have a polygenic origin too, such as in Rasmussen’s encephalitis [55] and in the mentioned cortical atrophy characterizing Alzheimer’s disease as well as several other brain conditions.

## 6. Genetic Versus Environmental Influence

**Figure 2:**
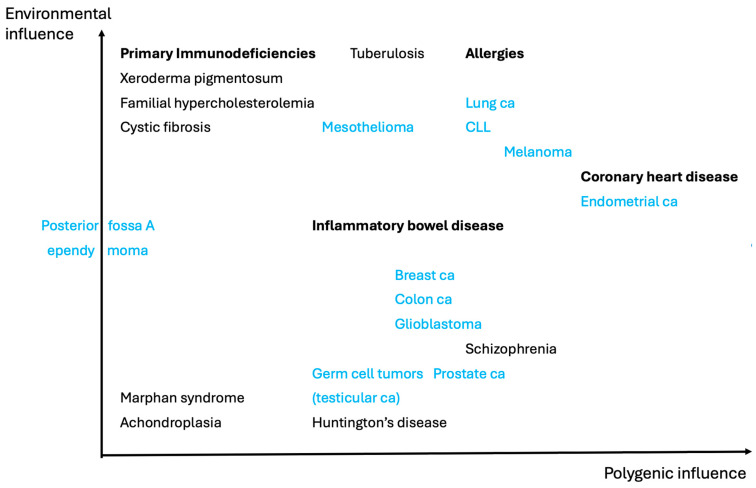
Influence of environmental elements on disease, from mono- to polygenic. Only selected disorders are given as examples. The positioning of diseases is tentative; thus, depending on the applied disease definition, their location may vary. Various forms of cancers can be influenced by environmental factors such as asbestos exposure causing mesothelioma [56], smoking resulting in lung cancer [57,58], and human papillomavirus inducing cervical cancer [24], not presented in the figure. Certain other forms of neoplasms belong to the only group of polygenic diseases for which an environmental influence may be negligible. Bold denotes groups of diseases, and blue color marks tumors. Abbreviations: ca, cancer; CLL, chronic lymphocytic leukemia.

It is well known that it is the combination of genetics and environmental cues that underlies the susceptibility to disease. Whenever diseases are discussed, the balance between these two parameters is crucial. In Figure 2, we have made an attempt to classify diseases, neoplasms as well as non-neoplasms, accordingly. It should, however, be emphasized that many contributing factors are likely unknown, meaning that the position of a disease in Figure 2 may change when more information becomes available. Below, some of the diseases in Figure 2 are described in more detail.

## 7. Classification of Autoimmune Diseases

An attempt to integrate multiple components for the establishment of taxonomy is represented by the recent report on inflammatory bowel disease (IBD) [59]. Based on genetics, syndromic features, effect of hematopoietic stem cell transplantation, proteomics, RNA- as well as single-cell RNA-sequencing of cell subsets from the intestine, the 102 monogenic disorders being most strongly associated with IBD were investigated. Biallelic defects of IL-10 signaling conferred the most pronounced susceptibility to monogenic IBD with onset in early infancy. Recently high titers of antibodies directed against IL-10 were shown to phenocopy the genetic disease [60], and this phenomenon is not unique to IL-10; other serum proteins can also serve as targets for an immune response, such as type I interferons in severe COVID-19 [61].

Genes whose expression is enriched in phagocytes were associated with impaired antimicrobial activity. Expression of genes affected in monogenic IBD showed enrichment in T regulatory (T_reg_) cells and inflammatory monocytes. In contrast, the corresponding result for genes involved in polygenic IBD was increased expression in enterocytes and mesenchymal cells. In rheumatoid arthritis (RA), related types of GWAS analyses conversely identified genes and pathways pinpointing CD4^+^ effector memory T (T_EM_) cells as important for pathogenesis [62].

While some autoimmune disorders are considered to be monogenic [63,64], the vast majority are polygenic, and many of the polymorphisms are related to factors protecting against infectious microorganisms [65]. In the main, the associated variants have small effect sizes. Significantly associated genes in autoimmunity primarily identify crucial immunological pathways [66]. In contrast to the above examples of IBD and RA, it is often unclear which cell type causes autoimmunity [64]. Like for many polygenic diseases, epigenetic changes, which become more pronounced with age, show an emerging role in autoimmunity [61].

## 8. Chronic Lymphocytic Leukemia—Genetic and Environmental Influence

Chronic lymphocytic leukemia (CLL) is the most common hematologic malignancy in adults in the Western world [67,68]. There are several predisposing genetic polymorphisms, and recently, 109 new variations were added, yielding a total of 202 candidate genetic Drivers, i.e., mutated genes being significantly overrepresented, of CLL by integrating data from 1148 patients [69]. Similar increments have been obtained for solid tumors when patient materials have been expanded, a recent example being the identification of many new Drivers based on more than 3000 patients with colorectal cancer [70,71].

As reviewed [72], in the late 1990s, CLL was subdivided into two disease subsets based on unmutated versus mutated immunoglobulin (IG) variable heavy chain genes (*IGVH*). This classical subdivision, which is still in use, reflects whether the malignant B lymphocytes have undergone the process of somatic hypermutation in lymph nodes or not (Figure 2). A decade ago, knowledge of the subsets was also highly clinically relevant because the mutational status also influenced the prognosis. However, with the therapeutic use of inhibitors for the cytoplasmic tyrosine kinase BTK, this has changed [73].

Knisbacher et al. [69] reported eight robust expression clusters strongly associated with *IGVH* mutational status, revealing new disease subtypes within the unmutated and mutated CLL subsets. In another recent large study, the genomic alterations instead defined a different set of CLL entities among the 485 studied patients [74]. Three subgroups represented patients with unmutated *IGVH* leukemia cells, one of which lacked a DNA damage response signature, whereas two represented mutated forms of *IGVH*. Thus, whereas some potential differences were noted between the two studies, together representing in excess of 1500 patients, combining these data sets would enable an even greater analytic depth. Based on these two reports, CLL could be classified into several subgroups, even if not yet into a novel, purely genetic variant-centered nosology. We suggest that a related taxonomy could likely be applied to other hematological malignancies as well.

Moreover, the discovery of stereotyped, (quasi)identical B cell receptor (BCR) immunoglobulins shared by different patients with CLL suggests the presence of common antigens in CLL pathogenesis [75]. Stereotyped BCR was initially described in 2003, when CLL cases with BCRs encoded by the *IGHV3-21* gene were found to carry highly homologous or even identical amino acid sequences within the variable heavy complementarity determining region 3 (VH CDR3) [76]. Moreover, they exhibited very restricted usage of the *IGLV3-21* gene. Subsequent studies corroborated that a large fraction of CLL patients can be assigned to different disease subsets based on distinct, stereotyped variable heavy complementarity determining region 3 (VH CDR3) sequences [77,78]. Stereotypy between geographically distant and unrelated patients implies that CLL ontogeny is not stochastic but rather related to common antigenic determinants. Stereotypy extends to shared somatic mutations, similar genetic and epigenetic profile of the CLL clones and functional responses, and also to similar clinical outcomes [79]. After analyzing almost 30,000 different CLL patients [78], 41% were found to express stereotyped BCRs, which is remarkable. Thus, the corresponding, calculated, random stereotype frequency is in the range of 1:10^−16^ to 1:10^−18^ [80]. Collectively, these observations provide strong molecular evidence for antigen selection in the ontogeny for this large CLL subgroup. When the total number of CLL patients reaches 100,000, it is projected that close to 100% of patients will express stereotyped BCRs (Kostas Stamatopoulos, personal communication). 

Concerning taxonomy, a comparison between the *International Consensus* and the *Fifth WHO* classifications for CLL shows that the diagnostic criteria are the same [81]. Mutation status of *IGHV* and *TP53*/17p should be assessed, whereas the situation for other frequently mutated genes remains optional. Thus, the mutational spectrum is beginning to have an influence, but, as yet, only in a minor way. Integrating both the intrinsic genetic alterations and stereotypy may also give us insight into the fundamental question: is CLL driven primarily by intrinsic genomic alterations or rather by cancer cell interactions with their microenvironment via specific BCR subsets?

In contrast, in atopic dermatitis, there was an increased usage of certain *IGHV* genes in IgE-producing cells, but there were no public *IGHE* clones among atopic dermatitis patients. This pattern and the degree of somatic hypermutation differed from both healthy controls and patients with psoriasis [82]. Given the strong influence of the environment for the development of CLL, it seems likely that many other immune system disorders, which are not known to be environment-dependent, are also strongly influenced by such cues.

## 9. Treatments Directed Against Drivers

The response to treatment is a way to identify functional Drivers, which is important for tumor classification. Drugs directed against various mutated forms of the small GTPase RAS, such as sotorasib, [83] target a signaling molecule whose gene is very frequently mutated in neoplasms. Another example is the use of tyrosine kinase inhibitors, which are directed against either Drivers or non-mutated signaling molecules to which tumors are addicted, such as BTK in hematological malignancies, the first targeting BTK being an irreversible binder named ibrutinib [84]. To this end, the first FDA (the United States Food and Drug administration) approved, target-directed analysis used for selecting tumor treatment was in breast cancer 25 years ago. Immunohistochemical analysis of the receptor tyrosine kinase ERBB2 (HER2) was here applied to direct the use of the monoclonal antibody trastuzumab. In 2022, there were 27 approved companion biomarkers [85].

An extreme therapeutic use of genetic gain-of-function markers is to select treatment only based on the molecular findings and *irrespective* of the origin of the tumor. This is the case for specific alterations such as *NTRK* gene fusions, *BRAF* gene mutations causing substitution of valine by glutamic acid at amino acid 600 (V600E), and *RET* gene fusions [86]. Moreover, additional genetic markers such as microsatellite instability and high mutational burden are also used in this context [85]. Thus, therapy to inactivate the effects of Driver mutations is expected to be target- rather than cell-origin-dependent.

## 10. Susceptibility to Infections and Secondary Illness

COVID-19 has resulted in a unique global research effort with >420,000 entries in PubMed to date, using ‘COVID-19’ as a search term, making it the most studied viral infection. The corresponding figure for ‘influenza’ yielded > 160,000 entries. What about the putative factors influencing the susceptibility to severe COVID-19? Apart from age, which is the single strongest predictor of severity, several genetic components also play decisive roles [61]. Genome-scale, CRISPR-induced, loss-of-function screens have recently uncovered rare genetic variants whose loss confers *resistance* to SARS-CoV-2 viral infection [87]. Of note, such protective traits are very difficult to identify in population studies for statistical reasons.

Based on the general knowledge on viruses as obligate intracellular pathogens relying on the host machinery for their replicative cycle, multiple cellular components are needed. The defense against SARS-CoV-2 and other viruses relies on the initial innate immune response followed by adaptive immunity. The interferon system comprises a large set of members which act together with intracellular signaling molecules, and jointly they control hundreds of interferon-responsive genes [88]. Concerted efforts have uncovered a number of single-gene defects causing life-threatening infection with SARS-CoV-2, many of which encompass interferon and interferon-related genes [89]. These also include *TLR7* and *IRF7* loss-of-function variants, which cause a lack of functional plasmacytoid dendritic cells (pDCs). These cells are crucial for localizing to SARS-CoV-2-infected areas, where they secrete very large amounts of type I and III (λ) interferons [90,91]. In other diseases, such as hematological malignancies, prone to severe COVID-19, the pDC population may instead be *numerically* compromised, as observed in hematological malignancies [92].

A potent polygenic contribution has also been shown for susceptibility to grave COVID-19 [61,93,94,95]. Interestingly, genes inherited from Neanderthals confer both increased sensitivity to [96] and protection against [97] severe COVID-19.

Also, for other infectious diseases, such as tuberculosis, many genetic elements are known to control predisposition [98]. Moreover, infections may secondarily induce cancer. This is especially true for chronic infections, an example being hepatitis B virus (HBV). HBV is known to annually cause millions of cases of liver cancer, with cirrhosis predisposing to tumor development as reviewed [99,100,101]. Multiple components contribute to the outcome of both hepatitis B and C virus infections, including host, viral, and environmental factors [98].

A prime example of infectious agents causing disease is Epstein–Barr virus, a ubiquitous oncogenic virus that is associated with a number of different human malignancies, such as lymphoma, nasopharyngeal carcinoma, and gastric cancer, as well as autoimmune disorders, including multiple sclerosis [102]. Upon infection, EBV establishes life-long latency in humans, and its viral proteins and non-coding RNAs induce EBV-mediated disease pathologies. Human T-cell leukemia virus type 1 (HTLV-1), a human retrovirus, is associated with two distinct types of diseases: adult T-cell leukemia–lymphoma and a chronic inflammatory central nervous system disease [103]. It was the first human retrovirus to be associated with a human cancer. While the mechanisms differ among the above-mentioned disorders, it demonstrates the complexity of diseases induced by infectious agents for which a genetic influence is found both from the pathogen and from the host.

## 11. Genetic Disease Causing Susceptibility to Infection

In monogenic disease, a single gene outweighs the contribution of all other genetic elements and sometimes also that of environmental factors (Figure 2). However, monogenic disorders are also essentially always dependent on other genetic elements as well as on the environment. What is described below differs from the previous section in that multiple components from both the pathogen and the host interact. As an example, cystic fibrosis is mainly considered to be monogenic with full penetrance, but this is not always the case [104]. Moreover, the accompanying devastating lung injuries are only manifested after the exposure to *Pseudomonas aeruginosa* bacteria, which thrive in the dehydrated, highly viscous mucus. The same is true for essentially all primary immunodeficiencies; until birth, affected children are only very rarely exposed to infectious microorganisms and are therefore completely healthy. Different heterozygous mutations affecting the cystic fibrosis transmembrane conductance regulator (CFTR) also exacerbate the lung injuries in primary immunodeficiencies [105]. This suggests that haploinsufficiency may be the underlying mechanism.

## 12. Non-Infectious Environmental Cues, Modifier Genes, and the Susceptibility to Disease

Another example of a single gene outweighing the contribution of genetic and environmental influences is xeroderma pigmentosum, formed by a collection of functionally related diseases caused by different loss-of-function variants resulting in extreme sensitivity to ultraviolet irradiation. Avoiding exposure to sunlight protects against the severe skin disease, but a large proportion of patients also develop neurodegeneration as well as non-skin cancer unrelated to light [106].

Moreover, there are monogenic disorders for which there are no known environmental cues and no known modifier genes (Figure 2). Examples are the most common form of dwarfism (achondroplasia), caused by gain-of-function mutations in the *FGFR3* gene [107,108] and the *FBN1* variants found in Marfan syndrome [109]. It would, however, be extremely surprising if modifier genes did not exist also for these disorders.

A rather unique disease is the severe, pediatric hindbrain tumor *posterior fossa A ependymoma*, which seemingly even lacks a genetic component but carries chromatin alterations in the form of hypomethylation. This central nervous neoplasm, which thus is devoid of Driver mutations (Figure 2), has been proposed to be purely epigenetically driven through metabolic regulation under hypoxic conditions [110]. The influence of environmental cues for this process is currently unknown.

In the group of monogenic diseases with less than full penetrance, this very characteristic is evidence that they are not only dependent on a single gene defect. An extreme case would be when loss-of-function mutations and *downstream* gain-of-function mutations in a common pathway occur in the same patient. In such highly rare instances, it is likely that the gain-of-function phenotype would prevail. Certain resistance mutations are selected for in B lymphocyte malignancies during BTK inhibitor treatment [72,111]. Some of them cause constitutively active forms of BTK’s substrate phospholipase-Cγ2 (PLCG2). Patients with X-linked agammaglobulinemia (XLA), a disease resulting from loss-of-function mutations in the *BTK* gene, lack B lymphocytes and are prone to infections [84,112]. If such patients developed a digenic disease, simultaneously carrying a *PLCG2* gain-of-function variation [113], XLA is presumably not phenotypically manifested. To the best of our knowledge, such a rare digenic disease has never been reported.

Hence, while the term *monogenic* is practical for many purposes (Figure 2), there is likely no disease which is entirely independent of other genetic factors. There is also a considerable influence of environmental elements for a substantial part of all illnesses, although for certain disorders, including cancer, no obvious external influence is known (Figure 2). However, in general, the contribution of the environment for cancer development is huge and includes lifestyle factors, infections, irradiation, and exposure to occupational toxins [114]. Furthermore, all forms of cancer are dependent on the tumor microenvironment, and in some cases, the microenvironment seems to even be the major driving force causing tumors to evolve [115]. Moreover, the effect of certain external components is often secondarily manifested as genetic changes in the form of mutated Driver genes.

Three major mechanisms underlying cancer exist: heredity, environment, and the number of cell divisions [116]. The fraction of cancers that are avoidable if environmental risk factors are eliminated is debated [114,116,117,118]. Moreover, some tumors are virtually only dependent on the number of cell divisions, randomly enabling scarce Driver mutations, and with a minimal influence of the environment (Figure 2). This contrasts with polygenic, non-cancerous diseases, where it is expected that at least some of the multiple contributing genetic factors are significantly affected by the environment [119].

## 13. Digenic and Trigenic Diseases

Following monogenic disease, the next class would be digenic disorders, i.e., illnesses with a highly dominating effect of two genes, as originally hypothesized in 1962 [120]. Apart from the two examples provided above, another digenic disorder is idiopathic hypogonadotropic hypogonadism caused by mutations in the *FGFR1* and *NSMF* genes located on chromosomes 8p and 9q, respectively [121]. In 2019, a web-accessible database named DIDA (DIgenic diseases DAtabase) was established for such disorders [122].

In 2024, the rare digenic inheritance of combined hereditary spherocytosis type III and XLA was reported [123]. With a prevalence of 1/10^4^ and 1/2 × 10^6^, respectively, this yields an estimated combined prevalence of 1/2 × 10^9^, i.e., corresponding to a hyper-rare digenic disorder. Another form of digenic inheritance involving the *BTK* and the *IL12RB1* genes was also recently reported [124]. However, owing to that the two affected individuals were both females, and that the X-linked trait therefore was not manifested, it does not qualify as a digenic *disease*. In contrast to the family with hereditary spherocytosis and XLA, which would constitute *phenotype maintenance* [9] since the phenotype was maintained, had a male been affected by both *BTK* and the *IL12RB1* gene mutations, it would have qualified as a digenic disease with an expected *phenotype conversion* [9].

Thus, in our recent review on disease numbers ([9], see Figure 3) we provide an example of trigenic disease, i.e., individuals simultaneously carrying three different rare disease genes. In this particular case, each of the inherited defects causes a defined primary immunodeficiency. Based on both the expected infectious panorama and the cellular phenotypes, this combination is projected to result in a novel illness with a *unique* phenotype (referred to as *phenotype conversion*) [9]. Thus, whether concurrent diseases actually cause a distinct illness will depend on whether their pathological mechanisms interact (phenotype conversion) or not (phenotype maintenance). In the latter case, the phenotypes are added, while in phenotype conversion, a unique disease entity appears. The above-mentioned trigenic disorder would be extremely infrequent, in this case occurring with a calculated frequency of only 10^−16^, based on the prevalence of the individual diseases. This means that during the history of mankind, it is highly unlikely that any human being has been affected.

To this end, it has been estimated that the global, trigenic interaction network in yeast is 100 times as large as the corresponding digenic network [125]. Recently, a patient with a novel trigenic combination of *ADH5*/*ADGRV1*/*ALDH2* pathogenic variants was reported [126]. Interestingly, and relevant for the discussion on malignant versus non-cancerous disease, this trigenic combination caused two distinct diseases, although only *ADH5* and *ALDH2* may have influenced each other mechanistically. One was the premalignant myelodysplastic syndrome and the other the non-malignant Usher syndrome, which causes hearing loss and retinitis pigmentosa. Another recent example is the combination of variations in the *NFKB1*, *PIK3R1*, and *TNFRSF13B* genes, resulting in a novel combined immunodeficiency [127]. We are not aware of any description of tetra- or pentagenic disease, but they are expected to exist, although increasingly infrequently and likely all belonging to the group of hyper-rare diseases.

## 14. More than 10,000 Polymorphisms Influence Body Height

The most extreme reported case of polygenic *influence* to date is the description of genes affecting body height. Thus, the recently reported GWAS of 5.4 million individuals of diverse ancestries found 12,111 independent single-nucleotide polymorphisms (SNPs) being significantly associated with height. These were estimated to account for nearly all of the common SNP-based heritability among Europeans [128]. Of note is that although this map was saturated for Europeans, it only achieved 10–20% saturation of other ancestries, demonstrating that there likely are many additional influential SNPs worldwide.

While many rare disorders have been found, the vast majority likely remain unidentified [9,129,130,131]. Furthermore, for numerous disorders lacking a dominating underlying genetic variant, the combined contribution of a multitude of variations, each with a minor influence, is likely commonplace. Thus, there are already ample examples of this, such as cardiovascular disease [132], kidney disorders [133], and autoimmunity [64].

## 15. Polygenic Risk Scores

Genetic susceptibility and environmental cues, including lifestyle factors, are key ingredients of complex diseases. *Polygenic scores* or *polygenic risk scores* (PGSs/PRSs) are calculated as a weighted sum of the genome-wide risk variants carried by a specific individual for a particular heritable characteristic. This provides a quantitative estimate of the likelihood that the individual will phenotypically manifest a trait [134]. Despite their potential utility for disease prediction and prevention, PGSs/PRSs have notable limitations. However, it has been shown that multiple scores from different traits better predict outcomes, in general, than scores from a single trait [135]. Moreover, cohorts in GWASs are often more extreme compared to normal patient populations and often lack fine-mapping of causal variants. As detailed above, complex traits are typically influenced by thousands of variants. Each of them makes only a small contribution, which may also not be generalizable to other ancestries [58,136]. Notwithstanding these limitations PGSs/PRSs are increasingly being adopted and found useful for making predictions. They have also been applied for tumors, such as basal cell and squamous cell carcinoma [137] and colon cancer [138]. Recently, three partially overlapping breast cancer PRSs comprising up to 313 SNPs have been proposed for European-ancestry women by the Breast Cancer Association Consortium [139].

PGSs/PRSs are increasingly being applied in non-cancerous disorders such as cardiovascular disease [132], type 2 diabetes [140], and rheumatoid arthritis [141], as well as for heritable disease-predisposing behaviors such as tobacco and alcohol use [58]. Likewise, PGSs/PRSs can be calculated for infectious diseases and their secondary manifestations, including cancer. Examples include malaria [142], dengue fever [143], and hepatic cancer and cirrhosis development after an HBV infection [96]. Recent results related to PGSs suggest moving away from discrete genetic ancestry clusters towards the continuum of genetic ancestries [144]. Because technical aspects remain challenging, as do certain ethical and social issues, this prompted the recent development of guidance for the use of PGSs by the American Society of Human Genetics [145].

It should also be noted that cross-trait assortative mating erroneously can influence the outcome of GWASs [1,146]. This phenomenon occurs when someone scoring highly for one trait non-randomly mates with partners who score high, or low, for a separate trait.

## 16. Estimating the Number of Polygenic Diseases—The Influence of Taxonomy

It is known that there are numerous polygenic diseases, but how many are there? This query can be divided according to clinical pragmatism or theory. As mentioned above, for body height, as many as 12,111 independent SNPs were recently uncovered [128]. Body height can be measured for everyone, whereas variations in disease derive from much smaller cohorts.

Randomly combining 12,111 variations equals a factorial of 12,111, which is a number too large to present. However, these polymorphisms cannot be regarded as independent, since it is likely that some of them would yield overlapping phenotypes (*phenotype maintenance*) [9]. Randomly combining just 1% of 12,111 equals 121! = 8.09 × 10^200^. For comparison, the largest number with a name is *googol*, which corresponds to 10^100^ [147]. This means that 121! is even in excess of *googol* multiplied by itself. Extrapolating from the literature, it seems reasonable to assume that a multitude of complex diseases could be influenced by as many as 121 parameters, even if some of the variants may be extremely rare.

## 17. Oncogenes and Tumor-Suppressor Genes and Tumor Classification

With these prerequisites, what would seem like a proper disease taxonomy? In the case of cancer, we propose using the functional Drivers causing the neoplasm to genetically subgroup tumors (Table 1). For a cancer cell, the estimated sum of active oncogenic and tumor-suppressor mutations was proposed to be in the range of 2–10 [148,149,150], a number that varies among neoplasms of different origins. The age of onset for different cancers correlates with the number of Drivers involved [148]. Conversely, when neoplasms are caused by a single catastrophic event, first described in the form of chromothripsis [151,152], this induces tumors also in young individuals [153].

It is also important to recognize that Driver mutations often occur in the absence of cancer, demonstrating that such alterations are neither necessary nor sufficient for inducing tumor formation [154,155,156,157,158]. Thus, in 1987, Hecht et al. reported that translocations frequently found in leukemias were present in healthy donors [154], and many other researchers have made similar observations [155,156,157,158]. Moreover, the Driver concept is a theory, the ‘somatic mutation theory’, which is based on highly significant statistical calculations, but it does not exclude other interpretations [159,160]. An alternative is the ‘tissue organization field theory’, which says that cancer arises from the disruption of interactions with adjacent tissue, such as altered intercellular chemical signals, mechanical forces, and bioelectric changes [159]. Other explanations have also been brought forward, such as altered metabolism, including a significant influence by mitochondria [161].

Certain Drivers are pleiotropic, such as TP53 and regulators of splicing and of epigenetics [162]. Thereby, a single mutation can affect several of the eight parameters characterizing Drivers listed in the introduction [15] (Table 1). The more pleiotropic the effect of the Driver mutation, the fewer additional acquired variations are expected to be required for malignancy. While certain oncogenes and tumor-suppressor genes dominate in various cancer types, there are numerous alternative tumor-inducing mutations affecting functionally related genes, thereby considerably increasing the number of disease entities.

**Table 1:** Parameters affected by Driver gene mutations.

Type of Cancer ^a^	Sustained Proliferation ^b^	Evasion of Immune Recognition	Cell Death Resistance	Replicative Immortality	Altered Metabolism	Reference
HCC	*HRAS/NRAS*, *MAPK* *TP53*		*TP53*	*TERT*	*NFE2L2* (oxidative stress) *HBx* (DNA repair) *TP53* (DNA repair and more)	Zucman-Rossie et al., 2015 [163]
Colon	*APC*, *KRAS FBXW7*, *TP53*		*TP53*		*FBXW7* (DNA repair) *TP53* (DNA repair and more)	Iranzo et al., 2018 [148]
AML	*DNMT3A*, *FLT3 NPM1*, *IDH2*		*DNMT3A* *IDH2*		*IDH2*	Iranzo et al., 2018 [148]; Hormaechea-Agulla et al., 2021 [164]; Issa and DiNardo, 2021 [165]
Lung cancer	*EGFR*, *KRAS*	HLA polymorphism		*TERT*	*BRCA2* (DNA repair) *ATM* (DNA repair)	Long et al., 2022 [57]

^a^ Abbreviations: HCC, hepatocellular carcinoma; AML, acute myelogenous leukemia. ^b^ Sustained proliferation can be caused by activation of growth or removal of growth suppression.

Moreover, additional Drivers may appear during the development of resistance to treatment [166]. Here, it is also worth mentioning that synthetic lethality is suggested to be mainly polygenic as well as influenced by the environment [167,168]. Drivers are not randomly selected *for*, because they could induce synthetic lethality or be redundant. For example, there are many different DNA damage repair defects [169]. For transformation, it may suffice to acquire a single-form DNA repair defect causing increasing numbers of acquired oncogenic and tumor-suppressor gene mutations. However, when a combination would result in very severe genomic instability, this may be highly harmful to the cancer cell itself. Thus, several different DNA repair defects may be involved in tumorigenesis for a particular cancer type, but only certain genetically determined combined alterations may be tolerated in individual tumor cells.

## 18. The Goner Concept

The Goner concept [11,12] teaches that certain mutations or epigenetic changes are toxic to tumors, i.e., the affected cells ‘are gone’ because when such alterations occur, the corresponding neoplastic cell is, thereby, lost (Table 2). As much as Drivers are crucial ingredients in the genetics of tumors, so too would Goners be. Thus, cancers with a specific genetic/epigenetic setup likely only tolerate selected additional alterations. Similar to Drivers, Goners are expected to vary extensively among different tumor types owing to the fact that their damaging behavior is likely to be highly dependent on the cellular context. The very same gene could therefore act as a Driver in one type of neoplasm and serve as a Goner in another cancer form. Moreover, Goners could, but need not, belong to the groups of oncogenes or tumor-suppressor genes. In fact, the first Goner defined was activating mutations affecting PLCG2, which is not known to be transforming [11,12]. While the *PLCG2*-mutated leukemia cells were selected for in patients with CLL treated with inhibitors for BTK, the GoF alteration was at same time toxic, thereby resulting in reduced clone sizes compared to non-damaging resistance mutations affecting BTK itself. A major practical impact of Goners is that their identification would immediately suggest new treatments.

As described in Table 2, Goners come in two flavors GoF/copy number gain and the opposite LoF (includes copy number loss), both of which are not tolerated. Furthermore, the Goner could be genetic or epigenetic in origin. Even if the outcome of epigenetic changes in general is much less studied, the same principle would apply as for genetic changes. The susceptibility to damaging mutations in tumors is caused by derangements in this type of cancer, making it sensitive to such noxiousness. Synthetic lethality could be causative [167], but, as mentioned, the Goner concept is much broader, as exemplified by the GoF mutations affecting PLCG2. Thus, contrary to most of the current targeted tumor treatments which impair the activity of Drivers, therapies based on the Goner concept should instead aim to activate the corresponding effector. As an example, when activating RAS mutations are lacking in a tumor because they induce toxicity, an option would be to activate damaging RAS signaling in the neoplasm.

Conversely, when recessive LoF mutations in, e.g., tumor-suppressor genes are identified as Goners, i.e., when their loss is not tolerated by the cancer cells, it is not because they serve as Passengers but because their absence causes toxicity. Hence, when their deficit is noxious, turning off tumor-suppressor gene activity would induce toxicity and constitute the therapeutic concept. Thus, as an example, when mutations affecting the tumor-suppressor PTEN are lacking, treatment with PTEN inhibitors is expected to induce toxicity. When the loss of tumor-suppressor genes instead induces tumor formation, such as the deficit of the retinoblastoma (Rb) protein in retinoblastoma [170], therapeutically introducing the corresponding gene into tumors would be extremely challenging. Removing their activity, when they act as Goners, is likely much easier, since small molecules which are readily taken up by cells may be able to achieve this.

## 19. Driver and Tumor-Suppressor Genes

Regarding Divers, there are two types: (1) oncogenes yielding a gain of function and (2) mainly recessive tumor-suppressor genes. Thus, while the two-hit hypothesis has been a leading concept, partial inactivation of suppressors may also play a role [170,171,172]. A special case is when there are redundant tumor-suppressor genes [167,173,174]. Such genes may be paralogs and, when unlinked, the likelihood of inactivating both paralogs must correspond to an extremely infrequent event given that specific mutations are very rare [167]. Hence, this would mean that in the tumor cell trajectory, some, or all, of the four copies of such genes need to be affected to obtain a loss-of-function phenotype. Moreover, at one end of the spectrum, inactivating all four copies may only afflict one of the eight parameters identified by Hanahan and Weinberg [15]. Owing to the fact that specific mutations are rare, the frequency of cancers caused by errors impairing redundant tumor suppressors in a single preneoplastic cell must be extremely minor. Random base substitutions occur with a frequency of 1.3 × 10^−8^ per site per generation in humans [175], albeit the frequency varies considerably among different malignancies. While mutations of many sites could inactivate, and although this is not the only way to lose the function of a tumor-suppressor gene, it still means that having 3–4 such specific changes would be extremely rare. We therefore propose that this form of tumor preferentially appears late in life. Based on these estimates, fewer than 1 per 10^8^ individuals is expected to acquire such a combination, and therefore, such neoplasms would belong to the hyper-rare category [9]. In this context, it should also be mentioned that for complex, non-cancerous disorders, a recent article found an underappreciated complexity of inheritance patterns of multiple Mendelian variants, which cannot be adequately described by a conventional definition of dominant or recessive [176]. These novel findings likely further increase the estimated number of complex diseases.

## 20. Pathway-Centered Nosologies

In the case of non-cancerous, complex disease, there is perhaps a greater challenge to find a basis for taxonomy. However, it is likely that many different types of variations could affect the same cellular pathway. If such a route encompasses a number of components, for which functional alterations may occur, it is expected that each of them would yield related pathology. Hence, in line with the proposition of Wang et al. [177] based on the interpretation of microarray data, we find it conceivable that a taxonomy for polygenic diseases should primarily be subdivided according to the affected pathways. Wang et al. named the uridine-5-diphosphate (UDP)–glycotransferase and the O-glycan biosynthesis pathways as two examples. From a theoretical point of view, it remains an open question whether the total number of different combinations of individual affected genes would serve as the preferred basis for enumerating how many diseases there actually are. Furthermore, a subdivision according to the affected pathway could also be of practical importance for treatment, since correcting a pathway may rectify a disease mechanism irrespective of the causative genetic variant. It is likely that for some complex disorders, a limited number of pathways induces abnormality, in its simplest form being digenic. Conversely, for others, it may be that it is the combination of multiple affected pathways which forms the underlying disease mechanism.

## 21. Discussion

In this *perspective* we establish that the estimated number of all polygenic disorders is very large but also highly influenced by the type of taxonomy applied. The mechanisms underlying polygenic diseases are complex, and over the last few years, a multitude of risk factors have been uncovered. These have been considered to mainly include genetic risk, but epigenetic changes have increasingly demonstrated their importance. However, epigenetic states are currently difficult to define and are therefore more cumbersome to include in disease classifications. Unlike monogenic disorders, for polygenic disease, the scientific community has not yet arrived at classifications taking the genetic or epigenetic influence into full account. For many types of neoplasms, the taxonomy even remains based only on non-genetic criteria developed decades ago, such as tumor location, the presence of metastasis, histology, and certain biomarkers. We propose, as a first step, to complement this clinical classification with information about the causative Drivers. Moreover, as new knowledge is acquired, the relevance of such descriptive, non-genetic parameters should be re-evaluated to further improve nosology. In parallel, functionally crucial alterations conditional on mutation-independent, epigenetic changes may become part of future disease classifications.

Estimating how many non-cancerous, polygenic diseases there are, based only on the actively contributing genetic polymorphisms, arrives at a clinically unmanageable number of unique combinations. This would represent the ultimate form of precision medicine. However, while difficult to turn into clinical practice, the genetic variants are useful for determining risk scores. For the clinical taxonomy of non-cancerous disorders, we therefore propose using the affected cellular pathways as the basis for disease subdivision. This could also pave the way for precision therapy, using the same drug to treat different illnesses affecting the same biological pathway. It is not yet possible to derive at a pathway-centered nosology for cancers, but this may eventually be the case.

In contrast, for the *theoretical* estimation of the total number of human complex diseases, the situation is different. Here, figures based on any unique combination of *relevant* genetic polymorphisms, or maintained *functionally relevant*, non-genetically derived epigenetic changes, would seem relevant. Many such unique combinations are projected to belong to the group referred to as hyper-rare, i.e., affecting fewer than 1 per 10^8^ individuals [9]. For neoplasms, an illustrative example of hyper-rarity would be those caused by abnormalities in redundant tumor-suppressor genes. Because mutations affecting a particular gene are rare, this would mean that in addition to the first mutation inactivating redundant tumor suppressors, at least two additional specific deleterious mutational events would be needed. Thus, together with the other genetic variations jointly leading to the transformation, this scenario would call for such a large number of alterations that the likelihood of their co-occurrence in a single cell would be extremely low. In a previous *perspective,* we demonstrated that by taking only monogenic disorders and environmental influences into account, there is an essentially infinite number of possible diseases [9]. We here argue that the same is true for polygenic diseases.

## Figures and Tables

**Table 2:** The Goner concept.

Gene	Mutation in Tumor
**Driver**	
Oncogene	Heterozygous GoF * or copy number gain
Tumor suppressor	Homozygous LoF ^#^
**Goner ^&^**	
For the tumor a noxious gene (e.g., a toxic oncogene)	Heterozygous GoF * mutation, or copy number gain, *lacking* in tumor because it induces toxicity in the affected cell ^€^
A gene which causes toxicity *when lost* (e.g., a tumor suppressor)	Homozygous LoF ^#^ *lacking* because the loss induces toxicity in the affected tumor cell ^€^
**Passenger**	
Random mutation	Acquired variation does not influence tumorigenicity

^&^ Goners are lost in tumors because they are noxious. The toxicity can be complete or partial, in which case tumor cells are not absent but reduced in number. The first example of a Goner was GoF * affecting PLCG2 [11,12], causing a hyperactive enzyme, not known to transform cells. While in the table only genetic alterations are presented, epigenetic changes are expected to have the same impact as either hetero- or homozygous changes. * GoF = gain of function by activating mutations or by gene amplification. Usually, this is a heterozygous alteration, but in rare cases, it could be homozygous. ^#^ LoF = loss of function by gene deletion or by another inactivating mutation. ^€^ The Goner concept means for certain genes, including oncogenes, that GoF mutations are not tolerated, i.e., they are gone in the corresponding tumors. Conversely, regarding LoF mutations, in the absence of certain genes acting recessively, including tumor-suppressor genes, the cancer cell does not thrive.

## References

[1] Fisher R.A. (1918). XV—The correlation between relatives on the supposition of Mendelian inheritance. Trans. R. Soc. Edinb..

[2] Boveri T. (1914). Zur Frage der Entstehung Maligner Tumoren.

[3] Hujoel M.L., Sherman M.A., Barton A.R., Mukamel R.E., Sankaran V.G., Terao C., Loh P.-R. (2022). Influences of rare copy-number variation on human complex traits. Cell.

[4] Bailey M.H., Tokheim C., Porta-Pardo E., Sengupta S., Bertrand D., Weerasinghe A., Colaprico A., Wendl M.C., Kim J., Reardon B. (2018). Comprehensive characterization of cancer genes and mutations. Cell.

[5] Sherman M.A., Yaari A.U., Priebe O., Dietlein F., Loh P.-R., Berger B. (2022). Genome-wide mapping of somatic mutation rates uncovers drivers of cancer. Nat. Biotechnol..

[6] Ng P.K.-S., Li J., Jeong K.J., Shao S., Chen H., Tsang Y.H., Sengupta S., Wang Z., Bhavana V.H., Tran R. (2018). Systematic Functional Annotation of Somatic Mutations in Cancer. Cancer Cell.

[7] Jensson B.O., Arnadottir G.A., Katrinardottir H., Fridriksdottir R., Helgason H., Oddsson A., Sveinbjornsson G., Eggertsson H.P., Halldorsson G.H., Atlason B.A. (2023). Actionable genotypes and their association with life span in Iceland. N. Engl. J. Med..

[8] McKusick V.A. (1969). On lumpers and splitters, or the nosology of genetic disease. Perspect. Biol. Med..

[9] Smith C.I.E., Bergman P., Hagey D.W. (2022). Estimating the number of diseases—The concept of rare, ultra-rare, and hyper-rare. iScience.

[10] Helleday T., Eshtad S., Nik-Zainal S. (2014). Mechanisms underlying mutational signatures in human cancers. Nat. Rev. Genet..

[11] Smith C.I.E., Zain R. (2024). Reduced clone size upon BTK inhibitor resistance mutations relates to toxicity caused by inherited *PLCG2* gain-of-function variations. Eur. J. Haematol..

[12] Bunz F. (2024). Passengers, drivers, and “goners”. Int. J. Cancer.

[13] Parsons D.W., Jones S., Zhang X., Lin J.C.-H., Leary R.J., Angenendt P., Mankoo P., Carter H., Siu I.-M., Gallia G.L. (2008). An integrated genomic analysis of human glioblastoma multiforme. Science.

[14] Versteeg R. (2014). Cancer: Tumours outside the mutation box. Nature.

[15] Hanahan D., Weinberg R.A. (2011). Hallmarks of cancer: The next generation. Cell.

[16] Hanahan D. (2022). Hallmarks of Cancer: New Dimensions. Cancer Discov..

[17] Fuxe J., Karlsson M.C. (2012). TGF-β-induced epithelial-mesenchymal transition: A link between cancer and inflammation. Semin. Cancer Biol..

[18] Hay E.D. (1985). Matrix-cytoskeletal interactions in the developing eye. J. Cell. Biochem..

[19] Yang Y., Jobin C. (2020). A mutational signature that can be made by a bacterium arises in human colon cancer. Nature.

[20] Huang S. (2012). Tumor progression: Chance and necessity in Darwinian and Lamarckian somatic (mutationless) evolution. Prog. Biophys. Mol. Biol..

[21] Waddington C.H. (1942). Canalization of development and the inheritance of acquired characters. Nature.

[22] Flavahan W.A., Gaskell E., Bernstein B.E. (2017). Epigenetic plasticity and the hallmarks of cancer. Science.

[23] Ho M., Patel A., Goh C.Y., Moscvin M., Zhang L., Bianchi G. (2020). Changing paradigms in diagnosis and treatment of monoclonal gammopathy of undetermined significance (MGUS) and smoldering multiple myeloma (SMM). Leukemia.

[24] McBride A.A., Warburton A. (2017). The role of integration in oncogenic progression of HPV-associated cancers Alison A. PLoS Pathog..

[25] Ly K.I., Blakeley J.O. (2019). The Diagnosis and Management of Neurofibromatosis Type 1. Med. Clin. N. Am..

[26] Flynn E.D., Lappalainen T. (2022). Functional Characterization of Genetic Variant Effects on Expression. Annu. Rev. Biomed. Data Sci..

[27] Lappalainen T., MacArthur D.G. (2021). From variant to function in human disease genetics. Science.

[28] Oliva M., Demanelis K., Lu Y., Chernoff M., Jasmine F., Ahsan H., Kibriya M.G., Chen L.S., Pierce B.L. (2022). DNA methylation QTL mapping across diverse human tissues provides molecular links between genetic variation and complex traits. Nat. Genet..

[29] Mostafavi H., Spence J.P., Naqvi S., Pritchard J.K. (2023). Systematic differences in discovery of genetic effects on gene expression and complex traits. Nat. Genet..

[30] Amariuta T. (2023). The power paradox of detecting disease-associated and gene-expression-associated variants. Nat. Genet..

[31] Choudhury A., Magill S.T., Eaton C.D., Prager B.C., Chen W.C., Cady M.A., Seo K., Lucas C.-H.G., Casey-Clyde T.J., Vasudevan H.N. (2022). Meningioma DNA methylation groups identify biological drivers and therapeutic vulnerabilities. Nat. Genet..

[32] Zhou W., Dinh H.Q., Ramjan Z., Weisenberger D.J., Nicolet C.M., Shen H., Laird P.W., Berman B.P. (2018). DNA methylation loss in late-replicating domains is linked to mitotic cell division. Nat. Genet..

[33] Johnstone S.E., Gladyshev V.N., Aryee M.J., Bernstein B.E. (2022). Epigenetic clocks, aging, and cancer. Science.

[34] Casanova J.-L., Abel L. (2022). From rare disorders of immunity to common determinants of infection: Following the mechanistic thread. Cell.

[35] Berglöf A., Turunen J.J., Gissberg O., Bestas B., Blomberg K.E.M., Smith C.E. (2013). Agammaglobulinemia: Causative mutations and their implications for novel therapies. Expert Rev. Clin. Immunol..

[36] He S., Sharpless N.E. (2017). Senescence in Health and Disease. Cell.

[37] Fuchs Y., Steller H. (2015). Live to die another way: Modes of programmed cell death and the signals emanating from dying cells. Nat. Rev. Mol. Cell Biol..

[38] Ellis R.E., Yuan J.Y., Horvitz H.R. (1991). Mechanisms and functions of cell death. Annu. Rev. Cell Biol..

[39] Watanabe-Fukunaga R., Brannan C.I., Copeland N.G., Jenkins N.A., Nagata S. (1992). Lymphoproliferation disorder in mice explained by defects in Fas antigen that mediates apoptosis. Nature.

[40] Wu J., Wilson J., He J., Xiang L., Schur P.H., Mountz J.D. (1996). Fas ligand mutation in a patient with systemic lupus erythematosus and lymphoproliferative disease. J. Clin. Investig..

[41] Dhingra R., Rabinovich-Nikitin I., Rothman S., Guberman M., Gang H., Margulets V., Jassal D.S., Alagarsamy K.N., Dhingra S., Ripoll C.V. (2022). Proteasomal degradation of TRAF2 mediates mitochondrial dysfunction in doxorubicin-cardiomyopathy. Circulation.

[42] Caccamo A., Branca C., Piras I.S., Ferreira E., Huentelman M.J., Liang W.S., Readhead B., Dudley J.T., Spangenberg E.E., Green K.N. (2017). Necroptosis activation in Alzheimer’s disease. Nat. Neurosci..

[43] Lalaoui N., Boyden S.E., Oda H., Wood G.M., Stone D.L., Chau D., Liu L., Stoffels M., Kratina T., Lawlor K.E. (2020). Mutations that prevent caspase cleavage of RIPK1 cause autoinflammatory disease. Nature.

[44] Kaiser W.J., Daley-Bauer L.P., Thapa R.J., Mandal P., Berger S.B., Huang C., Sundararajan A., Guo H., Roback L., Speck S.H. (2014). RIP1 suppresses innate immune necrotic as well as apoptotic cell death during mammalian parturition. Proc. Natl. Acad. Sci. USA.

[45] Zhang T., Xu D., Liu J., Wang M., Duan L.-J., Liu M., Meng H., Zhuang Y., Wang H., Wang Y. (2023). Prolonged hypoxia alleviates prolyl hydroxylation-mediated suppression of RIPK1 to promote necroptosis and inflammation. Nat. Cell Biol..

[46] Gougeon M.-L., Garcia S., Heeney J., Tschopp R., Lecoeur H., Guetard D., Rame V., Dauguet C., Montagnier L. (1993). Programmed cell death in AIDS-related HIV and SIV infections. AIDS Res. Hum. Retroviruses.

[47] Klenk H.-D., Rott R. (1988). The molecular biology of influenza virus pathogenicity. Adv. Virus Res..

[48] Hernandez-Segura A., Nehme J., Demaria M. (2018). Hallmarks of cellular senescence. Trends Cell Biol..

[49] Birch J., Gil J. (2020). Senescence and the SASP: Many therapeutic avenues. Genes Dev..

[50] Kawamura Y., Oka K., Semba T., Takamori M., Sugiura Y., Yamasaki R., Suzuki Y., Chujo T., Nagase M., Oiwa Y. (2023). Cellular senescence induction leads to progressive cell death via the INK4a-RB pathway in naked mole-rats. EMBO J..

[51] McPherron A.C., Lawler A.M., Lee S.-J. (1997). Regulation of skeletal muscle mass in mice by a new TGF-beta superfamily member. Nature.

[52] Rodriguez J., Vernus B., Chelh I., Cassar-Malek I., Gabillard J.C., Sassi A.H., Seiliez I., Picard B., Bonnieu A. (2014). Myostatin and the skeletal muscle atrophy and hypertrophy signaling pathways. Cell. Mol. Life Sci..

[53] Sartori R., Romanello V., Sandri M. (2021). Mechanisms of muscle atrophy and hypertrophy: Implications in health and disease. Nat. Commun..

[54] Tadros R., Francis C., Xu X., Vermeer A.M.C., Harper A.R., Huurman R., Bisabu K.K., Walsh R., Hoorntje E.T., Rijdt W.P.T. (2021). Shared genetic pathways contribute to risk of hypertrophic and dilated cardiomyopathies with opposite directions of effect. Nat. Genet..

[55] Ai J., Wang Y., Liu D., Fan D., Wang Q., Li T., Luan G., Wang P., An J. (2021). Genetic Factors in Rasmussen’s Encephalitis Characterized by Whole-Exome Sequencing. Front. Neurosci..

[56] Klebe S., Leigh J., Henderson D.W., Nurminen M. (2019). Asbestos, Smoking and Lung Cancer: An Update. Int. J. Environ. Res. Public Health.

[57] Long E., Patel H., Byun J., Amos C.I., Choi J. (2022). Functional studies of lung cancer GWAS beyond association. Hum. Mol. Genet..

[58] Saunders G.R.B., Wang X., Chen F., Jang S.-K., Liu M., Wang C., Gao S., Jiang Y., Khunsriraksakul C., Otto J.M. (2022). Genetic diversity fuels gene discovery for tobacco and alcohol use. Nature.

[59] Bolton C., Smillie C.S., Pandey S., Elmentaite R., Wei G., Argmann C., Aschenbrenner D., James K.R., McGovern D.P., Macchi M. (2022). An integrated taxonomy for monogenic inflammatory bowel disease. Gastroenterology.

[60] Griffin H., Ceron-Gutierrez L., Gharahdaghi N., Ebrahimi S., Davies S., Loo P.S., Szabo A., Williams E., Mukhopadhyay A., McLoughlin L. (2024). Neutralizing Autoantibodies against Interleukin-10 in Inflammatory Bowel Disease. N. Engl. J. Med..

[61] Zhang Q., Bastard P., Cobat A., Casanova J.-L. (2022). Human genetic and immunological determinants of critical COVID-19 pneumonia. Nature.

[62] Okada Y., Wu D., Trynka G., Raj T., Terao C., Ikari K., Kochi Y., Ohmura K., Suzuki A., Yoshida S. (2014). Genetics of rheumatoid arthritis contributes to biology and drug discovery. Nature.

[63] Bigley T.M., Cooper M.A. (2021). Monogenic autoimmunity and infectious diseases: The double-edged sword of immune dysregulation. Curr. Opin. Immunol..

[64] Gerussi A., Soskic B., Asselta R., Invernizzi P., Gershwin M.E. (2022). GWAS and autoimmunity: What have we learned and what next. J. Autoimmun..

[65] Gutierrez-Arcelus M., Rich S.S., Raychaudhuri S. (2016). Autoimmune diseases—Connecting risk alleles with molecular traits of the immune system. Nat. Rev. Genet..

[66] Mazzone R., Zwergel C., Artico M., Taurone S., Ralli M., Greco A., Mai A. (2019). The emerging role of epigenetics in human autoimmune disorders. Clin. Epigenetics.

[67] Burger J.A. (2020). Treatment of Chronic Lymphocytic Leukemia. N. Engl. J. Med..

[68] Campo E., Jaffe E.S., Cook J.R., Quintanilla-Martinez L., Swerdlow S.H., Anderson K.C., Brousset P., Cerroni L., de Leval L., Dirnhofer S. (2022). The International Consensus Classification of Mature Lymphoid Neoplasms: A report from the Clinical Advisory Committee. Blood.

[69] Knisbacher B.A., Lin Z., Hahn C.K., Nadeu F., Duran-Ferrer M., Stevenson K.E., Tausch E., Delgado J., Barbera-Mourelle A., Taylor-Weiner A. (2022). Molecular map of chronic lymphocytic leukemia and its impact on outcome. Nat. Genet..

[70] Cornish A.J., Gruber A.J., Kinnersley B., Chubb D., Frangou A., Caravagna G., Noyvert B., Lakatos E., Wood H.M., Thorn S. (2024). The genomic landscape of 2,023 colorectal cancers. Nature.

[71] Nunes L., Li F., Wu M., Luo T., Hammarström K., Torell E., Ljuslinder I., Mezheyeuski A., Edqvist P.-H., Löfgren-Burström A. (2024). Prognostic genome and transcriptome signatures in colorectal cancers. Nature.

[72] Smith C.I.E., Burger J.A. (2021). Resistance Mutations to BTK Inhibitors Originate From the NF-κB but Not From the PI3K-RAS-MAPK Arm of the B Cell Receptor Signaling Pathway. Front. Immunol..

[73] Burger J.A., Barr P.M., Robak T., Owen C., Ghia P., Tedeschi A., Bairey O., Hillmen P., Coutre S.E., Devereux S. (2019). Long-term efficacy and safety of first-line ibrutinib treatment for patients with CLL/SLL: 5 years of follow-up from the phase 3 RESONATE-2 study. Leukemia.

[74] Robbe P., Ridout K.E., Vavoulis D.V., Dréau H., Kinnersley B., Denny N., Chubb D., Appleby N., Cutts A., Cornish A.J. (2022). Whole-genome sequencing of chronic lymphocytic leukemia identifies subgroups with distinct biological and clinical features. Nat. Genet..

[75] Stamatopoulos K., Agathangelidis A., Rosenquist R., Ghia P. (2016). Antigen receptor stereotypy in chronic lymphocytic leukemia. Leukemia.

[76] Tobin G., Thunberg U., Johnson A., Eriksson I., Söderberg O., Karlsson K., Merup M., Juliusson G., Vilpo J., Enblad G. (2003). Chronic lymphocytic leukemias utilizing the VH3-21 gene display highly restricted Vλ2-14 gene use and homologous CDR3s: Implicating recognition of a common antigen epitope. Blood.

[77] Agathangelidis A., Darzentas N., Hadzidimitriou A., Brochet X., Murray F., Yan X.-J., Davis Z., Van Gastel-Mol E.J., Tresoldi C., Chu C.C. (2012). Stereotyped B-cell receptors in one-third of chronic lymphocytic leukemia: A molecular classification with implications for targeted therapies. Blood.

[78] Agathangelidis A., Chatzidimitriou A., Gemenetzi K., Giudicelli V., Karypidou M., Plevova K., Davis Z., Yan X.-J., Jeromin S., Schneider C. (2021). Higher-order connections between stereotyped subsets: Implications for improved patient classification in CLL. Blood.

[79] Ten Hacken E., Gounari M., Ghia P., Burger J.A. (2018). The importance of B cell receptor isotypes and stereotypes in chronic lymphocytic leukemia. Leukemia.

[80] Briney B., Inderbitzin A., Joyce C., Burton D.R. (2019). Commonality despite exceptional diversity in the baseline human antibody repertoire. Nature.

[81] Falini B., Martino G., Lazzi S. (2022). A comparison of the International Consensus and 5th World Health Organization classifications of mature B-cell lymphomas. Leukemia.

[82] Luo L., Luo Y., Xu J., Zhu R., Wu J., Liu X., Li W., Yao X. (2022). Heterogeneous origin of IgE in atopic dermatitis and psoriasis revealed by B cell receptor repertoire analysis. Allergy.

[83] Hong D.S., Fakih M.G., Strickler J.H., Desai J., Durm G.A., Shapiro G.I., Falchook G.S., Price T.J., Sacher A., Denlinger C.S. (2020). KRAS^G12C^ Inhibition with Sotorasib in Advanced Solid Tumors. N. Engl. J. Med..

[84] Smith C.I.E. (2017). From identification of the BTK kinase to effective management of leukemia. Oncogene.

[85] Schilsky R.L., Longo D.L. (2022). Closing the Gap in Cancer Genomic Testing. N. Engl. J. Med..

[86] Cocco E., Scaltriti M., Drilon A. (2018). NTRK fusion-positive cancers and TRK inhibitor therapy. Nat. Rev. Clin. Oncol..

[87] Pairo-Castineira E., Rawlik K., Bretherick A.D., Qi T., Wu Y., Nassiri I., McConkey G.A., Zechner M., Klaric L., Griffiths F. (2023). GWAS and meta-analysis identifies 49 genetic variants underlying critical COVID-19. Nature.

[88] Daniloski Z., Jordan T.X., Wessels H.-H., Hoagland D.A., Kasela S., Legut M., Maniatis S., Mimitou E.P., Lu L., Geller E. (2021). Identification of Required Host Factors for SARS-CoV-2 Infection in Human Cells. Cell.

[89] Jones C.E., Tan W.S., Grey F., Hughes D.J. (2021). Discovering antiviral restriction factors and pathways using genetic screens. J. Gen. Virol..

[90] Asano T., Boisson B., Onodi F., Matuozzo D., Moncada-Velez M., Maglorius Renkilaraj M.R.L., Zhang P., Meertens L., Bolze A., Materna M. (2021). X-linked recessive TLR7 de-ficiencyin ~1% of men under 60 years old with life-threatening COVID-19. Sci. Immunol..

[91] Campbell T.M., Liu Z., Zhang Q., Moncada-Velez M., Covill L.E., Zhang P., Darazam I.A., Bastard P., Bizien L., Bucciol G. (2022). Respiratory viral infections in otherwise healthy humans with inherited IRF7 deficiency. J. Exp. Med..

[92] Smith C.I.E., Zain R., Österborg A., Palma M., Buggert M., Bergman P., Bryceson Y. (2022). Do reduced numbers of plasmacytoid dendritic cells contribute to the aggressive clinical course of COVID-19 in chronic lymphocytic leukaemia?. Scand. J. Immunol..

[93] Degenhardt F., Ellinghaus D., Juzenas S., Lerga-Jaso J., Wendorff M., Maya-Miles D., Uellendahl-Werth F., ElAbd H., Rühlemann M.C., Arora J. (2022). Detailed stratified GWAS analysis for severe COVID-19 in four European populations. Hum. Mol. Genet..

[94] Kousathanas A., Pairo-Castineira E., Rawlik K., Stuckey A., Odhams C.A., Walker S., Russell C.D., Malinauskas T., Wu Y., Millar J. (2022). Whole-genome sequencing reveals host factors underlying critical COVID-19. Nature.

[95] Ellinghaus D., Degenhardt F., Bujanda L., Buti M., Albillos A., Invernizzi P., Fernández J., Prati D., Baselli G., Severe COVID-19 GWAS Group (2020). Genomewide association study of severe COVID-19 with respiratory failure. N. Engl. J. Med..

[96] Zeberg H., Pääbo S. (2020). The major genetic risk factor for severe COVID-19 is inherited from Neanderthals. Nature.

[97] Zeberg H., Pääbo S. (2021). A genomic region associated with protection against severe COVID-19 is inherited from Neandertals. Proc. Natl. Acad. Sci. USA.

[98] McHenry M.L., Williams S.M., Stein C.M. (2020). Genetics and evolution of tuberculosis pathogenesis: New perspectives and approaches. Infect. Genet. Evol..

[99] Cai X., Zha H., Yang Z., Du Y., Dai X., Yang B., Wang J., He Q., Weng Q. (2022). Genetic dominance of transforming growth factor-β1 polymorphisms in chronic liver disease. Front. Immunol..

[100] Kanda T., Goto T., Hirotsu Y., Moriyama M., Omata M. (2019). Molecular Mechanisms Driving Progression of Liver Cirrhosis towards Hepatocellular Carcinoma in Chronic Hepatitis B and C Infections: A Review. Int. J. Mol. Sci..

[101] Tandoh K.Z., Quaye O. (2022). Genetic associations in chronic hepatitis B infection: Toward developing polygenic risk scores. Futur. Microbiol..

[102] Damania B., Kenney S.C., Raab-Traub N. (2022). Epstein-Barr virus: Biology and clinical disease. Cell.

[103] Miura M., Naito T., Saito M. (2022). Current perspectives in human T-cell leukemia virus type 1 infection and Its associated diseases. Front. Med..

[104] Boussaroque A., Audrézet M.-P., Raynal C., Sermet-Gaudelus I., Bienvenu T., Férec C., Bergougnoux A., Lopez M., Scotet V., Munck A. (2020). Penetrance is a critical parameter for assessing the disease liability of CFTR variants. J. Cyst. Fibros..

[105] Lawless D., Allen H.L., Thaventhiran J.E.D., Goddard S., Burren O.S., Robson E., Peckham D., Smith K.G.C., Savic S., NIHR BioResource–Rare Diseases Consortium (2023). Prevalence of CFTR variants in PID patients with bronchiectasis—An important modifying co-factor. J. Allergy Clin. Immunol..

[106] Kraemer K.H., DiGiovanna J.J., Tamura D., Adam M.P., Everman D.B., Mirzaa G.M., Pagon R.A., Wallace S.E., Bean L.J.H., Gripp K.W., Amemiya A. (2003). Xeroderma Pigmentosum. GeneReviews^®^ [Internet].

[107] Rousseau F., Bonaventure J., Legeai-Mallet L., Pelet A., Rozet J.-M., Maroteaux P., Le Merrer M., Munnich A. (1994). Mutations in the gene encoding fibroblast growth factor receptor-3 in achondroplasia. Nature.

[108] Shiang R., Thompson L.M., Zhu Y.Z., Church D.M., Fielder T.J., Bocian M., Winokur S.T., Wasmuth J.J. (1994). Mutations in the transmembrane domain of FGFR3 cause the most common genetic form of dwarfism, achondroplasia. Cell.

[109] Milewicz D.M., Braverman A.C., De Backer J., Morris S.A., Boileau C., Maumenee I.H., Jondeau G., Evangelista A., Pyeritz R.E. (2021). Marfan syndrome. Nat. Rev. Dis. Primers.

[110] Michealraj K.A., Kumar S.A., Kim L.J., Cavalli F.M., Przelicki D., Wojcik J.B., Delaidelli A., Bajic A., Saulnier O., MacLeod G. (2020). Metabolic Regulation of the Epigenome Drives Lethal Infantile Ependymoma. Cell.

[111] Woyach J.A., Furman R.R., Liu T.-M., Ozer H.G., Zapatka M., Ruppert A.S., Xue L., Li D.H.-H., Steggerda S.M., Versele M. (2014). Resistance Mechanisms for the Bruton’s Tyrosine Kinase Inhibitor Ibrutinib. N. Engl. J. Med..

[112] Vetrie D., Vorechovsky I., Sideras P., Holland J., Davies A., Flinter F., Hammarström L., Kinnon C., Levinsky R., Bobrow M. (1993). The gene involved in X-linked agammaglobulinaemia is a member of the src family of protein-tyrosine kinases. Nature.

[113] Zhou Q., Lee G.-S., Brady J., Datta S., Katan M., Sheikh A., Martins M.S., Bunney T.D., Santich B.H., Moir S. (2012). A hypermorphic missense mutation in PLCG2, encoding phospholipase Cg2, causes a dominantly inherited autoinflammatory disease with immunodeficiency. Am. J. Hum. Genet..

[114] Hoover R.N. (2000). Cancer—Nature, nurture, or both. N. Engl. J. Med..

[115] Hoogstrate Y., Draaisma K., Ghisai S.A., van Hijfte L., Barin N., de Heer I., Coppieters W., Bosch T.P.v.D., Bolleboom A., Gao Z. (2023). Transcriptome analysis reveals tumor microenvironment changes in glioblastoma. Cancer Cell.

[116] Tomasetti C., Vogelstein B. (2015). Variation in cancer risk among tissues can be explained by the number of stem cell divisions. Science.

[117] Tomasetti C., Li L., Vogelstein B. (2017). Stem cell divisions, somatic mutations, cancer etiology, and cancer prevention. Science.

[118] Tybjerg A.J., Friis S., Brown K., Nilbert M.C., Morch L., Køster B. (2022). Updated fraction of cancer attributable to lifestyle and environmental factors in Denmark in 2018. Sci. Rep..

[119] Balliu B., Carcamo-Orive I., Gloudemans M.J., Nachun D.C., Durrant M.G., Gazal S., Park C.Y., Knowles D.A., Wabitsch M., Quertermous T. (2021). An integrated approach to identify environmental modulators of genetic risk factors for complex traits. Am. J. Hum. Genet..

[120] Defrise-Gussenhoven E. (1962). Hypothèes de dimérie et de non-pénétrance. Acta Genet..

[121] Pitteloud N., Quinton R., Pearce S., Raivio T., Acierno J., Dwyer A., Plummer L., Hughes V., Seminara S., Cheng Y.-Z. (2007). Digenic mutations account for variable phenotypes in idiopathic hypogonadotropic hypogonadism. J. Clin. Investig..

[122] Gazzo A.M., Daneels D., Cilia E., Bonduelle M., Abramowicz M., Van Dooren S., Smits G., Lenaerts T. (2016). DIDA: A curated and annotated digenic diseases database. Nucleic Acids Res..

[123] Almutairy K.A., Alasmari B.G., Rayees S. (2024). Digenic Inheritance of Hereditary Spherocytosis Type III and X-linked Agammaglobulinemia: Coexistence of Two Distinct Recessive Disorders in a Male Child. Cureus.

[124] Kose H., Gorukmez O., Kilic S.S. (2024). Coexistence of IL12Rbeta1 and BTK Mutations in a Family. Immunology.

[125] Kuzmin E., VanderSluis B., Wang W., Tan G., Deshpande R., Chen Y., Usaj M., Balint A., Usaj M.M., van Leeuwen J. (2018). Systematic analysis of complex genetic interactions. Science.

[126] Kinoshita S., Ando M., Ando J., Ishii M., Furukawa Y., Tomita O., Azusawa Y., Shirane S., Kishita Y., Yatsuka Y. (2021). Trigenic ADH5/ALDH2/ADGRV1 mutations in myelodysplasia with Usher syndrome. Heliyon.

[127] Hargreaves C.E., Dhalla F., Patel A.M., de Oteyza A.C.G., Bateman E., Miller J., Anzilotti C., Ayers L., Grimbacher B., Patel S.Y. (2022). Resolving the polygenic aetiology of a late onset combined immune deficiency caused by NFKB1 haploinsufficiency and modified by PIK3R1 and TNFRSF13B variants. Clin. Immunol..

[128] Yengo L., Vedantam S., Marouli E., Sidorenko J., Bartell E., Sakaue S., Graff M., Eliasen A.U., Jiang Y., Raghavan S. (2022). A saturated map of common genetic variants associated with human height. Nature.

[129] Boycott K.M., Rath A., Chong J.X., Hartley T., Alkuraya F.S., Baynam G., Brookes A.J., Brudno M., Carracedo A., den Dunnen J.T. (2017). International cooperation to enable the diagnosis of all rare genetic diseases. Am. J. Hum. Genet..

[130] Smedley D., Smith K.R., Martin A., Thomas E.A., McDonagh E.M., Cipriani V., Ellingford J.M., Arno G., Tucci A., Vandrovcova J. (2021). 100,000 Genomes pilot on rare disease diagnosis in health care—Preliminary report. N. Engl. J. Med..

[131] Wainschtein P., Jain D., Zheng Z., Cupples L.A., Shadyab A.H., McKnight B., Shoemaker B.M., Mitchell B.D., TOPMed Anthropometry Working Group, NHLBI Trans-Omics for Precision Medicine (TOPMed) Consortium (2022). Assessing the contribution of rare variants to complex trait heritability from whole-genome sequence data. Nat. Genet..

[132] Aragam K.G., Jiang T., Goel A., Kanoni S., Wolford B.N., Atri D.S., Weeks E.M., Wang M., Hindy G., Zhou W. (2022). Discovery and systematic characterization of risk variants and genes for coronary artery disease in over a million participants. Nat. Genet..

[133] Khan A., Turchin M.C., Patki A., Srinivasasainagendra V., Shang N., Nadukuru R., Jones A.C., Malolepsza E., Dikilitas O., Kullo I.J. (2022). Genome-wide polygenic score to predict chronic kidney disease across ancestries. Nat. Med..

[134] Torkamani A., Wineinger N.E., Topol E.J. (2018). The personal and clinical utility of polygenic risk scores. Nat. Rev. Genet..

[135] Kullo I.J., Lewis C.M., Inouye M., Martin A.R., Ripatti S., Chatterjee N. (2022). Polygenic scores in biomedical research. Nat. Rev. Genet..

[136] (2022). Genetic futurism. Nat. Genet..

[137] Seviiri M., Law M.H., Ong J.-S., Gharahkhani P., Fontanillas P., Olsen C.M., Whiteman D.C., MacGregor S., 23andMe Research Team (2022). A multi-phenotype analysis reveals 19 susceptibility loci for basal cell carcinoma and 15 for squamous cell carcinoma. Nat. Commun..

[138] Chen X., Li H., Mandic M., Hoffmeister M., Brenner H. (2022). Assessment of body mass index, polygenic risk score, and development of colorectal cancer. JAMA Netw. Open.

[139] Jiao Y., Truong T., Eon-Marchais S., Mebirouk N., Caputo S.M., Dondon M.-G., Karimi M., Le Gal D., Beauvallet J., Le Floch É. (2022). Association and performance of polygenic risk scores for breast cancer among French women presenting or not a familial predisposition to the disease. Eur. J. Cancer.

[140] Hahn S.-J., Kim S., Choi Y.S., Lee J., Kang J. (2022). Prediction of type 2 diabetes using genome-wide polygenic risk score and metabolic profiles: A machine learning analysis of population-based 10-year prospective cohort study. EBioMedicine.

[141] Ishigaki K., Sakaue S., Terao C., Luo Y., Sonehara K., Yamaguchi K., Amariuta T., Too C.L., Laufer V.A., Scott I.C. (2022). Multi-ancestry genome-wide association analyses identify novel genetic mechanisms in rheumatoid arthritis. Nat. Genet..

[142] Ebel E.R., Uricchio L.H., Petrov D.A., Egan E.S. (2022). Revisiting the malaria hypothesis: Accounting for polygenicity and pleiotropy. Trends Parasitol..

[143] Pare G., Neupane B., Eskandarian S., Harris E., Halstead S., Gresh L., Kuan G., Balmaseda A., Villar L., Rojas E. (2020). Genetic risk for dengue hemorrhagic fever and dengue fever in multiple ancestries. EBioMedicine.

[144] Ding Y., Hou K., Xu Z., Pimplaskar A., Petter E., Boulier K., Privé F., Vilhjálmsson B.J., Loohuis L.M.O., Pasaniuc B. (2023). Polygenic scoring accuracy varies across the genetic ancestry continuum. Nature.

[145] Novembre J., Stein C., Asgari S., Gonzaga-Jauregui C., Landstrom A., Lemke A., Li J., Mighton C., Taylor M., Tishkoff S. (2022). Addressing the challenges of polygenic scores in human genetic research. Am. J. Hum. Genet..

[146] Border R., Athanasiadis G., Buil A., Schork A.J., Cai N., Young A.I., Werge T., Flint J., Kendler K.S., Sankararaman S. (2022). Cross-trait assortative mating is widespread and inflates genetic correlation estimates. Science.

[147] Kasner E., Newman J.R. (1940). Mathematics and the Imagination.

[148] Iranzo J., Martincorena I., Koonin E.V. (2018). Cancer-mutation network and the number and specificity of driver mutations. Proc. Natl. Acad. Sci. USA.

[149] Martincorena I., Raine K.M., Gerstung M., Dawson K.J., Haase K., Van Loo P., Davies H., Stratton M.R., Campbell P.J. (2018). Universal Patterns of Selection in Cancer and Somatic Tissues. Cell.

[150] Reiter J.G., Baretti M., Gerold J.M., Makohon-Moore A.P., Daud A., Iacobuzio-Donahue C.A., Azad N.S., Kinzler K.W., Nowak M.A., Vogelstein B. (2019). An analysis of genetic heterogeneity in untreated cancers. Nat. Rev. Cancer.

[151] Stephens P.J., Greenman C.D., Fu B., Yang F., Bignell G.R., Mudie L.J., Pleasance E.D., Lau K.W., Beare D., Stebbings L.A. (2011). Massive genomic rearrangement acquired in a single catastrophic event during cancer development. Cell.

[152] Leibowitz M.L., Zhang C.-Z., Pellman D. (2015). Chromothripsis: A New Mechanism for Rapid Karyotype Evolution. Annu. Rev. Genet..

[153] Arniani S., Pierini V., Pellanera F., Matteucci C., Di Giacomo D., Bardelli V., Quintini M., Mavridou E., Fernandez A.G.L., Nardelli C. (2022). Chromothripsis is a frequent event and underlies typical genetic changes in early T-cell precursor lymphoblastic leukemia in adults. Leukemia.

[154] Hecht F., Hecht B.K., Kirsch I.R. (1987). Fragile sites limited to lymphocytes: Molecular recombination and malignancy. Cancer Genet. Cytogenet..

[155] Ciwinska M., Messal H.A., Hristova H.R., Lutz C., Bornes L., Chalkiadakis T., Harkes R., Langedijk N.S.M., Hutten S.J., Menezes R.X. (2024). Mechanisms that clear mutations drive field cancerization in mammary tissue. Nature.

[156] Martincorena I., Fowler J.C., Wabik A., Lawson A.R.J., Abascal F., Hall M.W.J., Cagan A., Murai K., Mahbubani K., Stratton M.R. (2018). Somatic mutant clones colonize the human esophagus with age. Science.

[157] Yokoyama A., Kakiuchi N., Yoshizato T., Nannya Y., Suzuki H., Takeuchi Y., Shiozawa Y., Sato Y., Aoki K., Kim S.K. (2019). Age-related remodelling of oesophageal epithelia by mutated cancer drivers. Nature.

[158] Yizhak K., Aguet F., Kim J., Hess J.M., Kübler K., Grimsby J., Frazer R., Zhang H., Haradhvala N.J., Rosebrock D. (2019). RNA sequence analysis reveals macroscopic somatic clonal expansion across normal tissues. Science.

[159] Baker S.G. (2014). A cancer theory kerfuffle can lead to new lines of research. JNCI J. Natl. Cancer Inst..

[160] Baker S.G. (2021). The case for a cancer paradox initiative. Carcinogenesis.

[161] Seyfried T.N., Chinopoulos C. (2021). Can the Mitochondrial Metabolic Theory Explain Better the Origin and Management of Cancer than Can the Somatic Mutation Theory?. Metabolites.

[162] Boutelle A.M., Attardi L.D. (2021). p53 and Tumor Suppression: It Takes a Network. Trends Cell Biol..

[163] Zucman-Rossi J., Villanueva A., Nault J.-C., Llovet J.M. (2015). Genetic Landscape and Biomarkers of Hepatocellular Carcinoma. Gastroenterology.

[164] Hormaechea-Agulla D., Matatall K.A., Le D.T., Kain B., Long X., Kus P., Jaksik R., Challen G.A., Kimmel M., King K.Y. (2021). Chronic infection drives Dnmt3a-loss-of-function clonal hematopoiesis via IFNγ signaling. Cell Stem Cell.

[165] Issa G.C., DiNardo C.D. (2021). Acute myeloid leukemia with IDH1 and IDH2 mutations: 2021 treatment algorithm. Blood Cancer J..

[166] Ansari-Pour N., Samur M.K., Flynt E., Gooding S., Towfic F., Stong N., Estevez M.O., Mavrommatis K., Walker B., Morgan G.J. (2023). Whole-genome analysis identifies novel drivers and high-risk double-hit events in relapsed/refractory myeloma. Blood.

[167] Parrish P.C., Thomas J.D., Gabel A.M., Kamlapurkar S., Bradley R.K., Berger A.H. (2021). Discovery of synthetic lethal and tumor suppressor paralog pairs in the human genome. Cell Rep..

[168] Ryan C.J., Devakumar L.P.S., Pettitt S.J., Lord C.J. (2023). Complex synthetic lethality in cancer. Nat. Genet..

[169] Hopkins J.L., Lan L., Zou L. (2022). DNA repair defects in cancer and therapeutic opportunities. Genes Dev..

[170] Knudson A.G. (1971). Mutation and cancer: Statistical study of retinoblastoma. Proc. Natl. Acad. Sci. USA.

[171] Berger A.H., Knudson A.G., Pandolfi P.P. (2011). A continuum model for tumour suppression. Nature.

[172] Chen M., Zhang J., Berger A.H., Diolombi M.S., Ng C., Fung J., Bronson R.T., Castillo-Martin M., Thin T.H., Cordon-Cardo C. (2018). Compound haploinsufficiency of Dok2 and Dusp4 promotes lung tumorigenesis. J. Clin. Investig..

[173] Yoshihama Y., Namiki H., Kato T., Shimazaki N., Takaishi S., Kadoshima-Yamaoka K., Yukinaga H., Maeda N., Shibutani T., Fujimoto K. (2022). Potent and Selective PTDSS1 Inhibitors Induce Collateral Lethality in Cancers with PTDSS2 Deletion. Cancer Res..

[174] Zhao D., DePinho R.A. (2017). Synthetic essentiality: Targeting tumor suppressor deficiencies in cancer. BioEssays.

[175] Lynch M. (2010). Rate, molecular spectrum, and consequences of human mutation. Proc. Natl. Acad. Sci. USA.

[176] Heyne H.O., Karjalainen J., Karczewski K.J., Lemmelä S.M., Zhou W., Gen F., Havulinna A.S., Kurki M., Rehm H.L., Palotie A. (2023). Mono- and biallelic variant effects on disease at biobank scale. Nature.

[177] Wang K., Li M., Bucan M. (2007). Pathway-based approaches for analysis of genomewide association studies. Am. J. Hum. Genet..

